# Observations on Food Consumption Behaviors During the COVID-19 Pandemic in Oman

**DOI:** 10.3389/fpubh.2021.779654

**Published:** 2022-01-25

**Authors:** Tarek Ben Hassen, Hamid El Bilali, Mohammad S. Allahyari, Hazem Al Samman, Soroush Marzban

**Affiliations:** ^1^Program of Policy, Planning, and Development, Department of International Affairs, College of Arts and Sciences, Qatar University, Doha, Qatar; ^2^International Centre for Advanced Mediterranean Agronomic Studies (CIHEAM-Bari), Valenzano, Bari, Italy; ^3^Department of Agricultural Management, Rasht Branch, Islamic Azad University, Rasht, Iran; ^4^Faculty of Economic and Management Sciences, North-West University, Mmabatho, South Africa; ^5^Department of Finance and Economics, College of Commerce and Business Administration, Dhofar University, Salalah, Oman; ^6^Department of Agricultural Extension and Education, School of Agriculture, Shiraz University, Shiraz, Iran

**Keywords:** COVID-19, food behavior, food consumption, Oman, Gulf Cooperation Council (GCC)

## Abstract

This paper aims to study the perceptions of the impacts of the COVID-19 pandemic on behaviors related to diet and food shopping on a sample of 356 adults in Oman. The study is based on the results of an Arabic-language online survey conducted between September 15 and October 10, 2020, using the Survey Monkey platform. The questionnaire had 25 questions (multiple options and one option), subdivided into three parts. Respondents were asked to disseminate the survey to their networks as part of the study's snowball sampling method. Descriptive statistics and various statistical tests (e.g., U-Mann Whitney, Kruskal-Wallis, chi-square) have been used to evaluate the study results. The study showed a significant shift in the attitude and behavior of respondents regarding food and health. Indeed, the paper findings indicated (i) a shift to healthier diets, as shown by the fact that 45.5% of the participants increased their intake of fruits and vegetables, 42.4% ate more healthy foods, and 53.1% reduced their intake of unhealthy foods; (ii) an increase in the consumption of local products, owing to food safety concerns, with 25.8% of the cohort stating that they purchase more local food items; (iii) a shift in grocery shopping behaviors, especially with 28.1% of the participants buying more groceries online; (iv) the absence of panic buying in Oman, since 62.36% of the participants said they did not stockpile food items; and (v) a reduction of food waste. Indeed, 78.9% of the participants specified they were not wasting more food than average since the beginning of the pandemic, and 74.72% indicated they were more aware of how much food they were wasting. Surprisingly, COVID-19 appears to bring many beneficial adjustments in Oman to make food consumption more sustainable and healthier.

## Introduction

The COVID-19 epidemic created a global health crisis and became a challenge even to the most advanced health and governance systems in the world (1). Governments worldwide have contemplated various measures, such as school closure, lockdown, banning public events, and social distancing. While these efforts have been critical, many voices have pointed out their worrying psychological, social, and economic effects on global production and consumption systems (2). In the same line of rationale, COVID-19 has impacted agro-food systems at many levels, from farm to fork (3–8). Indeed, the pandemic had several impacts on diet and food behavior. Moreover, COVID-19 is a worldwide pandemic that created a global economic and financial crisis (2), which is expected to seriously affect food access, diet quality, and diversity (9, 10).

Firstly, consumers were worried about their families and the long-term prognosis during the start of the pandemic, so they concentrated on panic buying and stockpiling (11). Various episodes of panic buying of storable food products (e.g., pasta, rice, etc.) have been reported in several countries across the globe shortly after their first coronavirus cases were announced (10, 12–14).

Secondly, COVID-19 has altered people's eating patterns and dietary quality in many ways. COVID-19, on the one hand, triggered nutritional and health deterioration. The severe changes in lifestyle brought about by the lockdown/quarantine, as well as the broader situation, resulted in negative feelings such as boredom, depression, tension, and fear of the disease, which could alter diet, resulting in poor eating habits and frequent snacking (15). In several countries of the Gulf Cooperation Council (GCC) [viz. Bahrain, Kuwait, Oman, Qatar, Saudi Arabia, United Arab Emirates (UAE)], the pandemic aggravated existing prevalent obesity and overweight issues. Many researchers in the region highlighted that negative emotions resulting from the pandemic contributed to overeating, particularly of ‘comfort foods’ (e.g., chocolate) (16, 17). As observed globally, many consumers in the region developed a mechanism for dealing with negative moods via increasing their consumption of unhealthy, fatty, energy-dense foods (18).

On the other hand, COVID-19 forced people to reassess their habits, and many were more aware of their dietary habits (19). In Qatar (20) and Kuwait (21), people had cut down on unhealthy items, including fast food, cookies, cakes, and pastries. They also drank more water and ate more nutritious meals, including healthy snacks, fruits, and vegetables.

Third, COVID-19 has transformed people's food shopping habits (3). Given the perceived risk of shopping at a grocery store, consumers have decreased the number of grocery visits and purchased more on each visit to minimize their perceived risks of COVID-19 exposure (3, 22). Additionally, consumers turned to online shopping, which accelerated digital adoption and necessitated considerable changes to retail and commerce (23, 24). Since the pandemic outbreak, online shopping in the GCC area has seen tremendous development, as have local delivery applications (e.g., Talabat, Uber Eats, Instashop) (19). Also, online retail food products have experienced record growth, with delivery times ranging from two to 10 days, and minimum order amounts have been increased (25).

Nonetheless, the final COVID-19 findings may differ based on various circumstances, including epidemiological conditions, socio-economic development level, and the effectiveness of national health systems (4). In this regard, the example of Oman, a high-income country and one of the world's most food-secure nations, is particularly intriguing.

The Sultanate of Oman, one of the Gulf Cooperation Council (GCC) countries, covers 309,500 km^2^ and has a population of 4.6 million and a GDP per capita of 14971.7 US$ in 2019 (26). Despite substantial diversification efforts, oil is still the backbone of the Omani economy, constituting 70% of government revenues, 30% of the income, and more than 50% of exports in 2019. As a result, the country's budgetary situation is extremely vulnerable to oil prices fluctuations (27). In 2020, Oman's economy was projected to contract by 3.5% due to the twin effects of the rapid drop in oil prices and COVID-19. Consequently, it is expected that the deficit will rise to more than 17% of the GDP in 2020 (27). Oman documented its first case of COVID-19 on Feb 24, 2020 (28), and its first related death on Apr 1 (29). As of Apr 14, 2021, the Sultanate had 174,364 cases and 1,798 total deaths (30). Throughout the initial months of the pandemic, the Omani government adopted various measures to break the spread of COVID-19, such as lockdown, social distance, mobility restrictions, the prohibition of public gatherings, etc. (31, 32). These preventive actions have disturbed several sectors and posed various challenges (33). Likewise, these actions may have affected food consumption and food shopping behavior (11).

**Figure 1:**
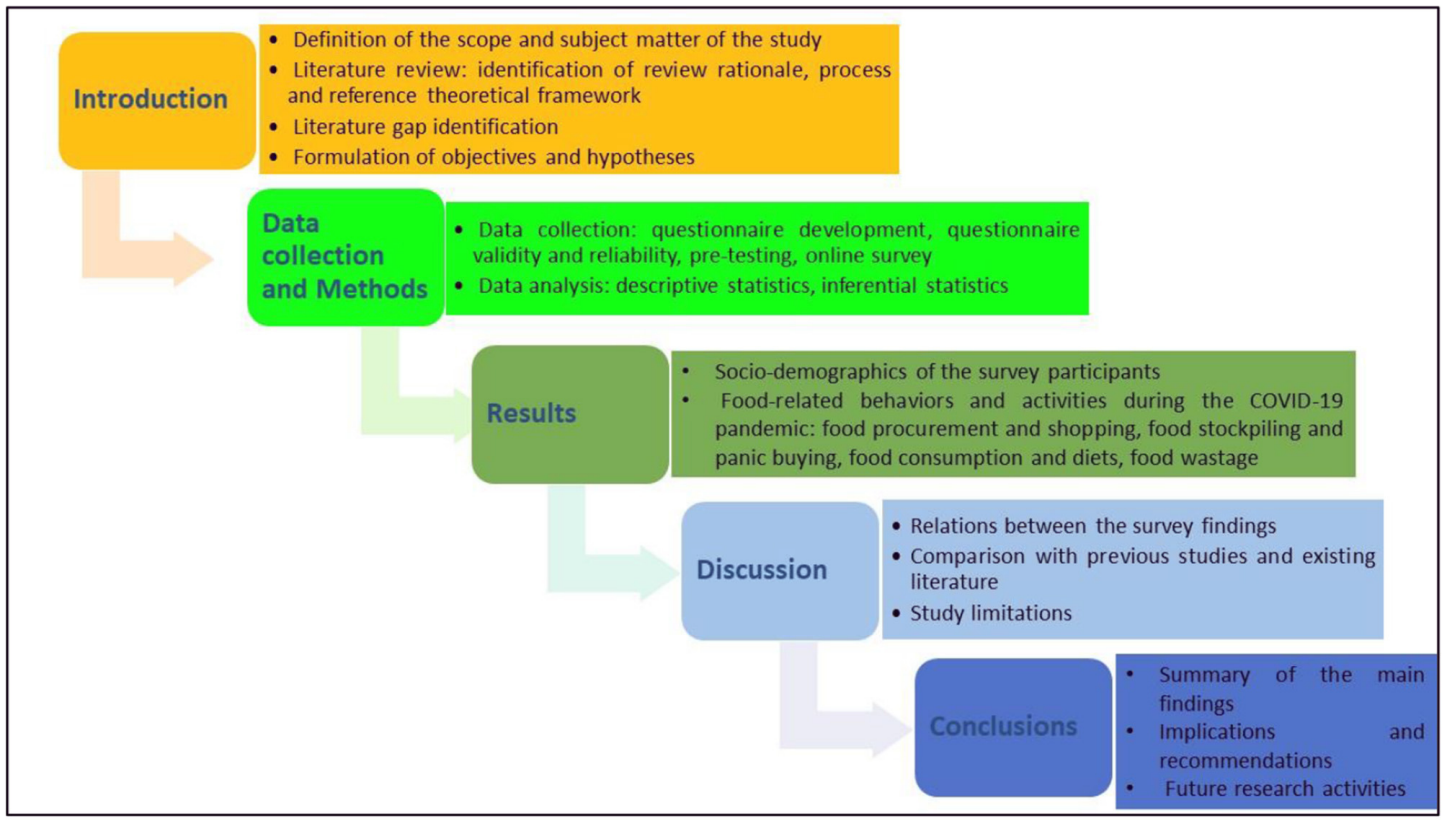
Research flowchart.

Accordingly, in this paper, a sample of 356 Omani adult consumers will be polled about their views on the potential consequences of the COVID-19 pandemic on their diet and food shopping behaviors. The research is based on four hypotheses: H1) the pandemic and the related negative feelings triggered a move toward unhealthy diet; H2) the pandemic caused a rise in online shopping; H3) the pandemic caused an increase in food stockpiling and panic buying; and H4) the pandemic caused an increase in food waste. Figure 1, informed by Ashraf et al. (34), depicts the organizational structure of the study.

## Data Collection and Methods

From September 15 to October 10, 2020, an online questionnaire^1^ in Arabic, Oman's official language, was administered using the Survey Monkey platform. The poll link was shared on social media such as Twitter and Facebook. The survey addresses the broad population of adults in Oman (those above the age of 18). The snowball sampling approach was utilized, and respondents were invited to share the online poll with their friends and relatives. We also opted for a non-probability sample technique, in which survey respondents were chosen at random and without reference to any prior criterion, except the age. In addition, there was no financial compensation for participating in the survey.

The Western Michigan University Human Subjects Institutional Review Board (HSIRB) approved all procedures involving research subjects following the Helsinki Declaration principles. At the start of the survey, all participants were told about the study goals. They supplied their digital permission concerning privacy and information management standards, as well as their confirmation that they were over the age of 18.

Many questions were raised in the research about the influence of the COVID-19 pandemic on food-related activities, such as food shopping, cooking, diet, and waste. The questionnaire was divided into three main parts, consisting of 25 different types of questions (multiple-choice, one option). (1) 10 questions on the social-demographic characteristics of the participants (e.g., education, gender, income, etc.); (2) 13 questions on food acquisition and diet (e.g., food purchases, food activities, food waste etc.) and 2 questions on emotions during the pandemic (see Appendix A).

The questionnaire was evaluated in two phases prior to release. Firstly, an expert panel performed a quality assessment of the content's validity to improve the research's validity and reliability. Inappropriate parts were removed based on professional evaluations, and the remaining items were altered to ensure accuracy and clarity. Secondly, a pre-test with 17 individuals was conducted to ensure the quality of the data. Before administering the survey, feedback was solicited in order to improve it. Finally, 356 valid responses were received. Further, the same questionnaire was utilized in prior surveys in several countries, such as Qatar (20), Lebanon (10), Serbia (13), Bosnia and Herzegovina (35), and Russia (22).

The survey findings were analyzed using the software SPSS (Statistical Package for Social Sciences) version 25.0. The descriptive statistics were computed (means, standard deviations, percentages, and frequencies). The percentages of answers and cases were determined via an examination of multiple responses. Non-parametric tests were utilized since the variables were nominal and ordinal. The U-Mann Whitney test was used for dichotomous, categorical independent variables (e.g., No = 0 / Yes = 1), while the Kruskal-Wallis test was employed to evaluate multiple-choice responses (e.g., occupation). Furthermore, the chi-square (2) test examined the connection between respondent variables and socio-demographic characteristics. The *p*-value for statistical significance was fixed at 0.05 for all tests.

## Results

### Study Participants' Social and Demographic Characteristics

The socio-demographic features of the respondents are shown in Table 1. The results indicated that 57.6% of the participants were men, 30.6% were married with children. Moreover, most respondents were middle-aged (58.4% were 25–45 years old), and 69.3% earned the same income as most of Oman's families. In general, the sample was well-educated, with 75.6% holding a Master's, university, or Ph.D. Only 23.3% had a high school diploma, and 1.12% were unqualified. Regarding occupation, 51.7% were working (full-time or part-time jobs), 32.8% were students, 9% were jobless and looking for employment, and 5.9% were homemakers (Table 1).

**Table 1:** Socio-demographic characteristics of the study participants (*n* = 356).

**Variable**		**Frequency**	**Valid percent**
Gender	Female	151	42.42
	Male	205	57.58
Age	18–24	117	32.87
	25–44	208	58.43
	45 and over	31	8.71
Level of education	No formal schooling or primary School	4	1.12
	Secondary School	83	23.31
	University Degree	224	62.92
	Higher Degree (MSc or PhD)	45	12.64
Income	Lower than most other households	37	10.4
	About the same as most other households	247	69.38
	Higher than other households	66	20.22
Occupation	In paid work (full time or part time)	184	51.69
	Student	117	32.87
	Unemployed and looking for work	32	8.99
	Home duties	21	5.90
	Retired/Age pensioner	2	0.56
Household composition	Single person household	4	1.12
	Living with parents	124	34.83
	Married with children	109	30.62
	Married without children	7	1.97
	Extended family	111	31.18
	Shared household, non-related	1	0.28

### Food-Related Behaviors and Activities During the COVID-19 Pandemic

The results indicated several modifications in participants' food shopping practices during the COVID-19 pandemic. Firstly, as shown in Table 2, 25.8% of the participants indicated that they purchased more local food items. Further, 28.1% specified that they purchased more groceries online, and 29.8% never did. Furthermore, 26.7% said they had more meals delivered to their homes from a typical restaurant or a fast-food restaurant or via a delivery app.

**Table 2:** Consumers' behavior trends during the COVID-19 pandemic (*n* = 356).

**Item**	**Percentage** *					**Mean**	VR**
	**Never**	**First Time**	**Less**	**About the same**	**More**		
Buying local food	9.55	3.37	10.95	43.82	25.84	3.63	0.56
Ordering groceries online	29.78	5.34	9.70	11.80	28.09	2.62	0.70
Buying food in person from a large supermarket	3.93	1.12	22.51	40.17	24.15	3.79	0.60
Having meals delivered directly to home from a full-service or fast food restaurant or by a delivery application	20.51	3.93	14.83	17.42	26.7	2.93	0.79

* *Scale: never = 0; first time = 1; less = 2; about the same = 3; more = 4*.

** *VR, Variance Ratio*.

Second, 84.2% of the participants said that they go shopping less often than customary, while 39.6% stated that they purchased more and much more quantity than usual on each shopping trip. Thirdly, as shown in Table 3, when asked about their diet during the COVID-19 pandemic, 54.5% of respondents indicated that they increased their water consumption, 45.5% increased their consumption of fruits and vegetables, and 42.4% increased their consumption of healthy foods (all by including “moderately more” and “much more”). In the meantime, 43% of the participants reduced their intake of unhealthy snacks, 53.1% consumed less unhealthy meals, and 35.6% consumed less packed frozen foods (all of these figures include “slightly less” and “much less”).

**Table 3:** Eating and drinking patterns during the COVID-19 pandemic (*n* = 356).

**Item**	**Percentage** *							**Mean**	VR**
	**Never**	**First Time**	**Much Less**	**Slightly Less**	**About the same**	**Moderately more**	**Much more**		
Water	0.84	0.56	1.97	2.25	39.89	23.31	31.18	4.74	0.60
Fruits/ Vegetables	0.84	1.40	4.78	4.78	42.70	29.78	15.73	4.39	0.57
Healthy foods	1.69	0.84	5.06	4.49	45.51	26.40	16.01	4.35	0.54
Healthy snacks	2.81	1.40	4.49	9.55	53.93	19.94	7.87	4.02	0.46
Candy, cookies, cakes, and pastries	2.53	1.69	14.61	21.63	38.76	11.80	8.99	3.64	0.61
Packaged frozen foods	10.67	1.69	15.73	19.94	37.92	11.24	2.81	3.18	0.62
Unhealthy snacks	8.43	2.25	22.47	20.51	31.74	10.67	3.93	3.13	0.68
Unhealthy foods (fast-food)	8.15	1.97	29.21	23.88	23.31	9.55	3.93	2.97	0.71
Canned food	12.92	2.81	21.07	19.10	30.90	10.11	3.09	2.95	0.69

* *Scale: never = 0; first time = 1; much less = 2; slightly less = 3; about the same = 4; moderately more = 5; much more = 6*.

** *VR, Variance Ratio*.

Fourthly, there have been some modifications in food-related activities. According to the findings, 56.1% of the cohort ate out less, and 44.6% ordered fewer take-out or fast food meals (all calculated by counting “slightly less” and “much less”). Moreover, 54.2% of those polled ate more with family members, 54.2% cooked and prepared food much more frequently, 46.3% cooked a lot, and 28.1% ate more between meals (e.g., snacks) (all calculated by counting “moderately more” and “much more”) (Table 4).

**Table 4:** Change of food-related activities during the COVID-19 pandemic (*n* = 356).

**Item**	**Percentage** *					**Mean**	VR**
	**Never**	**First Time**	**Less**	**About the same**	**More**		
Eating out	24.72	2.25	56.18	12.64	4.22	2.04	0.60
Ordering take-away or fast food meals with deliveries	19.94	2.53	44.66	15.45	17.42	2.65	0.74
Eating with family members	1.97	0.84	13.77	44.10	39.32	4.39	0.56
Cooking and preparing food	3.93	0.56	6.46	30.06	58.99	4.66	0.70
Spending a lot of time cooking	5.90	0.56	11.52	35.67	46.35	4.27	0.64
Eating between meals (e.g., snacks)	4.21	1.69	21.06	44.94	28.09	3.86	0.55
Making easy meals	9.27	2.53	22.47	34.55	31.18	3.64	0.65

* *Scale: never = 0; first time = 1; less = 2; about the same = 3; more = 4*.

** *VR, Variance Ratio*.

Another notable outcome is the low panic buying. In fact, 62.3% of the cohort said that they had not stored food since COVID-19 became serious in Oman. There has been a decrease in food waste, with 78.9% reporting that they were not wasting more food than usual due to COVID-19, and 74.7% reporting that they were more conscious of the amount of food they were throwing away (Table 5).

**Table 5:** Changes in food behavior during the COVID-19 pandemic (*n* =356).

**Item**	**Percentage**		**Mean**	SD*
	**Yes**	**No**		
Do you buy more food out of fear or anxiety?	32.58	67.42	1.67	0.47
Do you eat more food out of boredom?	29.50	70.50	1.71	0.46
Are you wasting more food than usual?	21.10	78.90	1.79	0.41
Are you more aware of how much food you waste?	74.72	25.28	1.25	0.44

* *SD, Standard Deviation*.

Nonetheless, there have been substantial correlations between the participant's citizenship and food stockpiling (chi-square test *p* < 0.05). Indeed, 64.7% of the Omani respondents and only 33.3% of the non-Omani indicated that they did not stock up food. Stocking up food by the non-Omani was mainly motivated by concerns about obtaining enough food and food prices rising (Table 6).

**Table 6:** Stocking up and food-related concerns during the COVID-19 pandemic and comparison between groups of citizenships.

**Item**	Scale*/Percentage					**Mean**	**S.D**.	**U Mann Whitney test -** **Citizenship**
	**Not at all**	**Less**	**Moderate**	**Much**	**Very much**			
Obtaining enough food	39.04	14.04	25.28	11.52	10.11	2.40	1.36	3082.50**
Obtaining a variety of food	38.76	16.29	24.72	14.89	5.34	2.32	1.27	3381.50*
Access to healthy and safe food	37.08	12.92	23.88	18.54	7.58	2.47	1.35	3466.50*
Food prices rising	19.94	14.89	30.06	17.42	17.70	2.98	1.35	3132.00**

* *Scale: Not at all = 1; Less = 2; Moderate = 3; Much = 4; Very much = 5*.

** p < 0.01, *p < 0.05.

Furthermore, according to Table 7, the findings revealed a low prevalence of negative emotions such as fear, anxiety, and depression. Indeed, 40% of respondents said they were not nervous at all, 42% said they were not depressed at all, and 43% said they were not sad at all. Meanwhile, 51.41% of the cohort reported feeling optimistic, and 36.72% declared feeling calm.

**Table 7:** Negative and positive emotions since the onset of COVID-19 (*n* = 356).

**Emotion Item**	**Percentage** *				**Mean**	VR**
	**Not at all**	**Less**	**Moderate**	**Much**		
Nervous	40.06	17.33	24.15	18.46	2.32	0.60
Worried	22.44	18.47	23.86	35.49	2.91	0.76
Depressed	42	19.70	17.70	20.6	2.27	0.58
Sad	43.06	18.70	18.41	19.83	2.27	0.57
Scared	27.68	24.29	19.21	28.81	2.66	0.72
Bored	20.11	14.16	23.80	41.93	3.16	0.72
**Total of negative emotions**					2.60	
Calm	14.69	18.36	30.23	36.72	3.10	0.70
Optimistic	9.89	13.56	25.14	51.41	3.51	0.67
Excited	22.44	19.32	32.39	25.85	2.76	0.67
Happy	18.47	19.60	32.39	29.54	2.88	0.77
**Total of positive emotions**					3.07	

* *Scale: Not at all = 1; Less = 2; Moderate = 3; Much = 4*.

** *VR, Variance Ratio*.

## Discussion

This paper examined the impacts of the COVID-19 pandemic on diet and food shopping behaviors in Oman based on the perspectives of 356 consumers who participated in this study. Since the outbreak of the COVID-19 pandemic started, we have seen a significant shift in respondents' food and health-related behavior and attitudes. There have been noticeable shifts in the ways how people eat, purchase, and interact with food. The findings revealed several significant consumer trends that have an impact on the diet and eating behavior of the study participants.

First, intakes of unhealthy foods such as sweets and junk food during the epidemic have been reduced by most respondents. Meanwhile, more fruit and vegetables have been consumed in a healthier diet. This created a favorable transformation compared to the pre-COVID 19 State toward better eating habits and may assist in achieving the nation's health and nutrition vision for 2050 (36). Indeed, ranked amongst the most developed countries globally, Oman has experienced a rapid socio-economic development process in the past fifty years. Therefore, the prevalence of over-nutrition and associated morbidities grows in the Sultanate. A survey of 2017, led by the Omani Ministry of Health, indicated that 69.3% of men and 63.3% of women were overweight or obese. It also outlined a sharp rise in adult obesity since 1991 (37). As in the whole Middle East region, Oman is also witnessing some of the highest rates of childhood obesity (38). As a result, there is a high burden of non-communicable diseases (NCDs), particularly type 2 diabetes and kidney and heart diseases, among the Omani population (36). Moreover, in 2017, a survey highlighted that 57.3% of women and 63.9% of men consumed <5 portions of fruit and/or vegetables per day. Further, Afshin et al. (39) highlighted high sodium consumption, trans fats, and sugar-sweetened beverages (SSBs) among the Omani population.

Second, as shown in multiple countries throughout the globe (11, 24), most participants' food buying habits have changed due to COVID-19. On the one hand, as more people shop online to escape congested supermarkets, the digitization of food retail is speeding fast. This supports a general trend in the GCC area, where online shopping has grown significantly since the pandemic's beginning (19, 20). At the same time, several respondents still bought food in person to check the quality and freshness of the items. Similarly, shopping at grocery stores became the only activity available, with most entertainment activities closed (shopping centers, movies, etc.).

Moreover, the pandemic has also affected people's shopping habits since supermarkets are seen as risky places where people are afraid to be near one another. As witnessed in several countries, COVID-19 was linked to fewer shopping trips and increased purchases per trip. In addition, due to food safety concerns, the consumption of local food products rose. Concerns about the transmission of the virus grew with the COVID- 19 pandemic and an increasing number of people want to know where their food originates from. A preference for local products was generated by the unfounded belief of consumers that imported items represent a safety concern. A locally produced item is thought to be handled fewer times and therefore has a higher perception of safety (40). It may also be related to the distributions of global food chains and the resulting lack in the provision stream of imported items. In fact, the pandemic and associated actions caused substantial distortions in the food supply chain via logistical interrupts and restricted access to markets for commodities (4).

Third, the vast majority of respondents did not stockpile food. This is owing to the limited dissemination of negative emotions such as fear, anxiety, and despair. Indeed, most study participants are less concerned about their families and long-term prospects than those in other countries (11). Stress, despair, and anxiety may cause panic purchasing and hoarding, which is a way for consumers to reclaim control over their product procurement (41). Indeed, stockpiling food does give people a sense of power and control (42). In several countries in the Middle East and North Africa (MENA) region, there was high dissemination of negative emotions and consequently a spread of stockpiling. For example, in Lebanon, according to Ben Hassen et al. (10), 60.9% of the respondents were feeling depressed, 66.3% were nervous, and 60.2% were sad. Meanwhile, they emphasized the prevalence of panic purchasing in Lebanon, with 73.13 percent of respondents reporting that they stocked up on food once COVID-19 became serious. Similarly, in Morocco, 52.65% of interviewees reported having stockpiled food since COVID-19 became serious (43). Indeed, there was a rush to Moroccan retailers just before the lockdown in March 2020, and demand for flour and grains skyrocketed. Moroccans were worried about the Coronavirus and stockpiling in massive quantities. As a result, food prices have risen (43). In Oman, the government took several initiatives to mitigate the consequences of the epidemic on food supplies. In Oman, 80% of the food consumed is imported. The epidemic, however, had little effect on food supplies or pricing. In 2019, Oman was ranked 46th among 113 countries in the Global Food Security Index (44). In April 2020, at the beginning of the COVID-19 pandemic, the Omani authorities rushed to maintain the strategic food stock. To strengthen the reserve stock of essential food commodities. Moreover, in October 2020, the Omani government announced that essential food items are exempted from the Value-Added Tax (VAT) to ensure that the tax does not increase inflation and living costs (45). Additionally, the Omani government adopted clear and intense communication strategies to reassure its citizens. For example, in March 2020, the general director of commercial operations at the Omani PASFR affirmed that “The authority has made full preparations to confront the Coronavirus pandemic and that the food stock situation is good and there is no concern in providing basic food commodities.” Also, the authorities made continuous efforts to monitor markets and regulate prices. For instance, the government developed a range of e-platforms to promote online sales of agricultural products (46).

We did detect specific differences between Omani and non-Omani responders, though. Non-Omani purchased more food than Omani respondents. The socio-economic characteristics of Oman could explain this. In 2018, foreign workers made up 86% of the entire workforce. The private sector employed 86% of all foreign employees in the same year. The number of foreign workers in Oman increased from the 2000's to 2016 but declined. Since 2017, the government has imposed Omanisation quotas and restrictions on hiring foreign workers in several sectors (47). As a result, non-Omanis are more concerned about losing their jobs or having their salaries reduced due to the COVID-19 epidemic. Indeed, in 2020, the drop in oil prices and the disruptions from COVID-19 placed unprecedented strain on Oman's economy. Real GDP decreased by 2.8% in 2020 (48).

Finally, the absence of panic buying resulted in decreased food waste. Furthermore, this positive change may suggest that most research participants have adopted various positive methods for the administration of food throughout the pandemic (e.g., greater pre-shop preparation, improved food storage, and innovative cooking/prep procedures), as seen in the UK (49). This is a positive change since food waste in Oman is a significant issue, where food is primarily wasted at the level of consumers (50). Indeed, according to the Food Waste Index Report 2021(51), In Oman, 95 kg/capita of food is wasted every year, compared to an average of 79 kg/capita/year for high-income countries. This shows a potential path toward a more sustainable behavior in food consumption. The COVID-19 pandemic has shown an improvement in food waste behavior in Oman, as shown by studies in various countries in the region, such as Qatar (20), Lebanon (10), Tunisia (52), and Morocco (43).

Nonetheless, some survey methodologies and instruments have some limitations that might impair the sample's representativeness. The most significant limitation of this study is likely to be its sample bias. Indeed, the survey participants were chosen at random and freely. Because the questionnaire was filled out by unpaid volunteers, only those who had a clear interest in the topic could participate (cf. self-selection of the sample). Consequently, our sample may not represent the whole population of Oman. For example, in our sample, those with a university degree were more likely to be included (75.5%). Accordingly, it is challenging to extrapolate survey findings to the whole Oman population because of this biased sample. This bias may lead as well to inaccuracy in the reported behaviors. In general, in surveys, low-educated people tend to be underrepresented (53). Many of the above limitations apply to computer-assisted web interviewing (CAWI), which is often deployed in survey research (54–56). However, face-to-face research is challenging to achieve because of the COVID-19 condition and social distancing measures, and online surveys became more practical. To our knowledge, this is the first research in Oman to examine the influence of COVID-19 on food consumption patterns.

## Conclusion

Through a cross-sectional online survey, this paper examined the perceptions of Omani consumers on the impacts of the COVID-19 pandemic on food-related behaviors. Overall, the survey findings indicate that the COVID-19 pandemic has improved Oman's transition to more sustainable and healthy consumption practices. The results led to the rejection of three hypotheses – since the pandemic and the related negative feelings did not trigger a move toward unhealthy diets (H1) and it did not cause either an increase in food stockpiling and panic buying (H3) or an increase in food waste (H4) – and the confirmation of only H2 hypothesis relating to the rise in online shopping. Positive developments include purchasing local foods, improving food shopping and procurement planning, healthier diets, and less household food waste. However, since the COVID-19 pandemic is still underway and given the study's limitations described above, the results need to be checked and investigated in the future through a more extensive sample. Moreover, the present cross-sectional survey results represent a good baseline for future longitudinal studies on how the pandemic has affected food-related behaviors in Oman. They also provide valuable insights to inform policies and strategies aiming at mitigating the impacts of the pandemic on food sustainability, food security, and nutrition in the Sultanate and other GCC countries. In crisis circumstances, such as the COVID-19, the pace of collecting and releasing knowledge is especially relevant. A minimal understanding of attitudes, values, information, and behaviors may help new research and strategies.

## Data Availability Statement

The raw data supporting the conclusions of this article will be made available by the authors, without undue reservation.

## Ethics Statement

The studies involving human participants were reviewed and approved by this study was performed in compliance with the Helsinki Declaration guidelines. All procedures relevant to study participants were approved by the Western Michigan University Human Subjects Institutional Review Board (HSIRB). Participation in the research was voluntary. The patients/participants provided their written informed consent to participate in this study.

## Author Contributions

TBH, HEB, and MSA: conceptualization, methodology, and formal analysis. SM: software and validation. TBH and HAS: investigation. MSA: data curation. TBH and HEB: writing—original draft preparation, writing—review and editing, and project administration. All authors have read and agreed to the published version of the manuscript.

## Funding

The publication of this article was funded by the Qatar National Library.

## Conflict of Interest

The authors declare that the research was conducted in the absence of any commercial or financial relationships that could be construed as a potential conflict of interest.

## Publisher's Note

All claims expressed in this article are solely those of the authors and do not necessarily represent those of their affiliated organizations, or those of the publisher, the editors and the reviewers. Any product that may be evaluated in this article, or claim that may be made by its manufacturer, is not guaranteed or endorsed by the publisher.

## Footnotes

^1^“The West Michigan University's Food Consumption Changes 2020 study (57) and the United Nations System Standing Committee on Nutrition's (UNSCN) COVID-19 Survey (58) guided our questionnaire”.
