# Supplementary material for: Observations on Food Consumption Behaviors During the COVID-19 Pandemic in Oman
Source: Front Public Health. 2022 Jan 25;9:779654. doi: 10.3389/fpubh.2021.779654 (PMC8821884; doi:10.3389/fpubh.2021.779654)
Supplement: Supplementary file 1 [file Table_1.pdf]

Appendix A: Questionnaire

**Section 1: Socio-demographics**	
1	Gender
2	Where do you live? Urban region/ Rural area
3	Nationality: Omani/Non Omani
4	Age
5	Level of education
6	How would you describe your household income compared to other households in your country?
7	Occupation
8	What is your household composition?
9	How many people are currently living in your home?
10	Have you lost your job or had any pay reduction in your salary due to COVID-19? Yes/No
**Section 2: Food buying and consumption behavior**	
11	Below is a list of food-related behaviors. Please indicate how that behavior has changed for you as a result of the coronavirus (COVID-19) becoming serious in your country (7-point response scale: never = 0; first time = 1; much less = 2; slightly less = 3; about the same = 4; moderately more = 5; much more = 6.)<br>• Buying local food (produced in your country)<br>• Ordering groceries online<br>• Buying food in person from a large supermarket<br>• Buying food in person from a small supermarket or grocery store<br>• Having meals delivered directly to my home
12	What has changed in your shopping behavior during the outbreak of COVID-19 and lockdown?<br>• I go shopping less than usual<br>• I go shopping like I used to<br>• I go shopping more than usual
13	What has changed in the extent of your purchase during the outbreak of COVID-19 and lockdown?<br>• I buy a lot more than usual<br>• I buy more than usual<br>• I buy as same as usual<br>• I buy less than usual<br>• I buy a lot less than usual
14	Since the coronavirus (COVID-19) became serious in your country, have you eaten or drunk more or less of the following foods? (7-point response scale)<br>• Fruits/ Vegetables<br>• Meat<br>• Healthy food

	• Unhealthy food (Fast food)<br>• Water<br>• Can<br>• Candy, Cookies, cakes, and pastries<br>• Healthy Snacks<br>• Unhealthy snacks<br>• Packaged frozen foods<br>• Canned food
15	Since the coronavirus (COV-19) became serious in your country, have you done more or less of the following food related activities than you used to? (7-point response scale)<br>• Eating at home alone<br>• Eating with family members<br>• Eating out (e.g. restaurants/cafeteria/fast food)<br>• Eating at someone else's place (e.g. family, friends)<br>• Ordering take-away or fast food meals with deliveries<br>• Cooking and preparing food<br>• Spending a lot of time cooking<br>• Making easy meals (e.g., instant foods, etc.)<br>• Eating between meals (e.g. snacks)
16	Have you stocked up on food and beverages because of the coronavirus (COVID-19)? Yes/No
17	What type of food you stocked up the most during the outbreak of COVID-19 and lockdown? (Please select all that apply)<br>• Cereals and their products (bread, rice, pasta, flour, etc.)<br>• Roots and tubers (potatoes, etc.)<br>• Legumes (e.g. peas, chickpeas)<br>• Sugar<br>• Oil<br>• Fruits and vegetables<br>• Meat and meat products<br>• Fish and seafood<br>• Milk and dairy products<br>• Canned food<br>• None
18	Since the COVID-19 outbreak, did you notice that any of these items is less available? (Please select all that apply)<br>• Cereals and products (bread, rice, pasta, flour, etc.)<br>• Roots and tubers (potatoes, etc.)<br>• Legumes (e.g. peas, chickpeas)<br>• Sugar<br>• Oil<br>• Fruits and vegetables<br>• Meat and meat products<br>• Fish and seafood

	• Milk and dairy products<br>• Canned food<br>• None
19	Since the COVID-19 outbreak, did you notice any price increase for any of these items? (Please select all that apply)<br>• Cereals and products (bread, rice, pasta, flour, etc.)<br>• Roots and tubers (potatoes, etc.)<br>• Legumes (e.g. peas, chickpeas)<br>• Sugar<br>• Oil<br>• Fruits and vegetables<br>• Meat and meat products<br>• Fish and seafood<br>• Milk and dairy products<br>• Canned food<br>• None
20	How does stocking up on items make you feel? (5-point response scale: 1 (not at all),…5 (very much))<br>• Stocking up on items makes me feel less anxious<br>• Stocking up on items makes me feel more secure<br>• Stocking up on items comforts me<br>• Stocking up on items gives me a sense of control
21	Please indicate how concerned you have been since COVID-19 became serious in your country about the following food-related issues? 5-point response scale: 1 (not at all),…5 (very much))<br>• Obtaining enough food<br>• Obtaining a variety of food<br>• Access to healthy and safe food<br>• Food prices rising<br>• Food spreading COVID-19
22	Regarding changes in your food related behaviors since the outbreak of COVID-19: Yes/No<br>• Do you buy more food out of fear or anxiety?<br>• Do you eat more food out of fear, anxiety or boredom?<br>• Are you wasting more food than usual?<br>• Are you more aware of how much food you waste?
23	How has your food wastage changed during the outbreak of COVID-19 and lockdown?<br>• It has become much less<br>• Less<br>• Has not changed<br>• More<br>• Much more

Section 3: Emotions	
**24**	**Please indicate your negative feelings since the onset of COVID-19 (5-point response scale: 1 (not at all),…5 (very much))**<br>• Nervous<br>• Worried<br>• Depressed<br>• Sad<br>• Scared<br>• Bored
**25**	**Please indicate your positive feelings since the onset of COVID-19 (5-point response scale: 1 (not at all),… 5 (very much))**<br>• Calm<br>• Optimistic<br>• Excited<br>• Happy
